# Does treatment of adolescent depression reduce risk of later psychosis: A quasi-experimental study of selective serotonin reuptake inhibitor treatment in a total population cohort

**DOI:** 10.1192/j.eurpsy.2025.10050

**Published:** 2025-06-25

**Authors:** Colm Healy, Kirsite O’Hare, Ulla Lång, Martta Kerkelä, Jonah Byrne, Juha Veijola, Anna Pulakka, Johanna Metsälä, Eero Kajantie, Ian Kelleher

**Affiliations:** 1 Centre for Clinical Brain Sciences, Division of Psychiatry, University of Edinburgh, Edinburgh, UK; 2 School of Medicine, University College Dublin, Dublin, Ireland; 3 Faculty of Medicine, University of Oulu, Oulu, Finland; 4 Population Health Unit, Finnish Institute for Health and Welfare, Helsinki, Finland; 5 Research Unit of Population Health, University of Oulu, Oulu, Finland; 6 Clinical Medicine Research Unit, MRC Oulu, University of Oulu and Oulu University Hospital, Oulu, Finland; 7 Department of Clinical and Molecular Medicine, Norwegian University of Science and Technology, Trondheim, Norway; 8 St John of God Hospitaller Services Group, Dublin, Ireland

**Keywords:** depression, prevention, psychosis, schizophrenia, SSRIs, emulated target trial

## Abstract

**Background:**

Psychotic disorders are frequently preceded by depressive disorders, and it has been hypothesized that treatment of depression in youth may reduce risk for later psychosis. Using quasi-experimental methods, we estimated the causal relationship between the treatment of adolescent depression with selective serotonin reuptake inhibitors (SSRIs) and the risk of later psychosis.

**Methods:**

We used data linkage from multiple national Finnish registries for all individuals (*n* = 697,289) born between 1987 and 1997 to identify depression diagnosed before age 18, cumulative SSRI treatment within three years of diagnosis, and diagnoses of non-affective psychotic disorders by end of follow-up (age 20–29). We used instrumental variable analyses, exploiting variability in prescribing across hospital districts to estimate causal effects. Analyses were conducted using two-stage least squares modelling. Sensitivity analyses examined effects stratified by confounders and effects of specific SSRIs.

**Results:**

Our final sample included 22,666 individuals diagnosed with depression in adolescence, of whom 60.2% (*n* = 13,650) had used SSRIs. 10.7% of adolescents with depression went on to be diagnosed with a non-affective psychotic disorder. SSRI treatment for adolescent depression was not associated with a reduced risk of developing a psychotic disorder (one-year *β =* 0.04,CI:−0.01 to 0.09; two-years *β =* 0.02,CI:−0.06 to 0.09; three-years *β =* −0.02,CI:−0.08 to 0.05).

**Conclusions:**

Our quasi-experimental investigation does not support the hypothesis that treatment of adolescent depression reduces the subsequent risk of psychosis. Our findings question the assumption that treatment of common mental health disorders in youth may impact the risk of developing severe mental illnesses in adulthood.

Psychotic disorders, such as schizophrenia, are among the most disabling and economically expensive chronic medical conditions [1]. Prediction and prevention of psychosis has been a major focus of psychiatric research over the past two decades [2–4]. Psychotic disorders are frequently preceded by other mental health disorders in youth [5–8]. For example, in a national population study, we previously showed that up to half of all cases of psychotic disorders occurred in individuals who had attended child and adolescent psychiatry services at some point (age <18 years), most frequently for depressive disorders [6]. Interventions for adolescent depression, then, might be an important focus for the prevention of later severe mental illness.

Researchers have proposed that antidepressant medication may reduce the risk of psychotic disorders [9]. Not only are depressive disorders the most frequent mental health condition to precede the onset of schizophrenia [6, 7], cognitive models of mental illness also purport that low mood plays a causal role in the development of psychosis [10, 11]. Researchers have suggested that antidepressant medication may have preventative effects against the development of psychosis in a number of ways, including (1) by their effects on improving mood and reducing faulty (negative) appraisals of early (subclinical) psychotic symptoms, (2) by effects on neurochemical pathways that mediate stress responses, and (3) by preventing adverse mental health responses to environmental stressors [10].

The idea that treating depression in adolescence may prevent the onset of severe mental illness in adulthood is heuristically appealing – the so-called “intervention as prevention” hypothesis [12]. There is currently a lack of evidence, however, as to whether treatment of adolescent depression impacts the risk of subsequent severe mental illness, such as schizophrenia-spectrum disorders.

Typical observational analyses are unable to provide accurate evidence on the relationship between depression treatments and the risk of later psychosis. There is a relationship between the severity of depressive disorders and the likelihood of selective serotonin reuptake inhibitor (SSRI) treatment. Similarly, the severity of depressive disorders is related to the risk of later psychosis [13, 14]. Thus, a simple observational analysis might demonstrate an apparent relationship between SSRI treatment and increased psychosis risk, but this would be biased by the reason for treatment (severity of the depressive disorder, i.e., confounding by indication).

Ideally, the question of whether SSRI treatment in adolescence can reduce the risk of subsequent psychosis would be answered with a well-powered randomized controlled trial (RCT) following young people from adolescence into adulthood. However, such an RCT would have ethical and practical problems. It would be unethical to randomly withhold (in the control arm) an evidence-based medication treatment from young people with depressive disorders for several years in order to examine for a hypothetical reduction in psychosis risk. Furthermore, adolescents treated with antidepressants would need to be followed for many years to surpass the median age of psychosis onset (age 25 years) [15], making an RCT unfeasible.

Where a full-scale RCT is not ethical or feasible, quasi-experimental methods can be employed to help answer causal questions. An instrumental variable (IV) approach, such as using natural variation in prescribing practices between different physicians or clinical centers, can be used to investigate such questions [16, 17]. For example, Wang and colleagues used variability in antipsychotic medication prescriptions and demonstrated that treating older adults with conventional antipsychotic medication was associated with an elevated risk of death relative to atypical medication [18]. Other psychiatric-related investigations, exploiting the variability in treatment practices, have also been conducted [19–21].

The most common pharmacological treatment for depressive disorders, both in adolescence and in adulthood, is selective serotonin reuptake inhibitors (SSRIs). The rate of prescribing of SSRI treatment, however, has been shown to vary widely between treatment centers [22]. This variability provides an opportunity for a quasi-experimental design, which allows for causal estimation [23]. Specifically, it allows for the estimation of the causal effect of SSRI treatment for depression on the long-term risk of psychosis.

We applied an IV approach to a total population Finnish register linkage study, identifying all individuals born from 1987 to 1997 who attended child and adolescent psychiatry services and were diagnosed with a depressive disorder. We used variation in prescribing practice across hospital districts as an IV in order to investigate the potential causal relationship between SSRI treatment in adolescence and subsequent risk of psychotic disorder.

## Materials and methods

### National Registry Data

Finnish national registry data were used to identify the population of interest. We linked data from the Medical Birth Registry (birth records), the Care Registry for Health Care (medical diagnosis), Statistics Finland (and education records), Digital and Population Data Services (emigration record and death record), and the Social Insurance Institution of Finland (Kela, for medication purchase and reimbursement records). The medical reimbursement records from Kela were subsequently linked with the Finnish Medicines Agency medical product registry (Fimea) using the Nordic product number. This was used to identify the dose and packet size of the individual reimbursement. The Care Registry for Health Care provides information on all inpatient and outpatient visits to secondary-level healthcare from 1998 to the present. The registry has been shown to capture diagnoses of mental disorders with high validity [24–26]. The Kela registry provides information on social reimbursements, including medical reimbursements, on all individuals in Finland, regardless of age, wealth, or address [27]. The national health insurance scheme in Finland covers some of the cost of medicines prescribed by physicians [27].

### Participants

**Figure 1.**
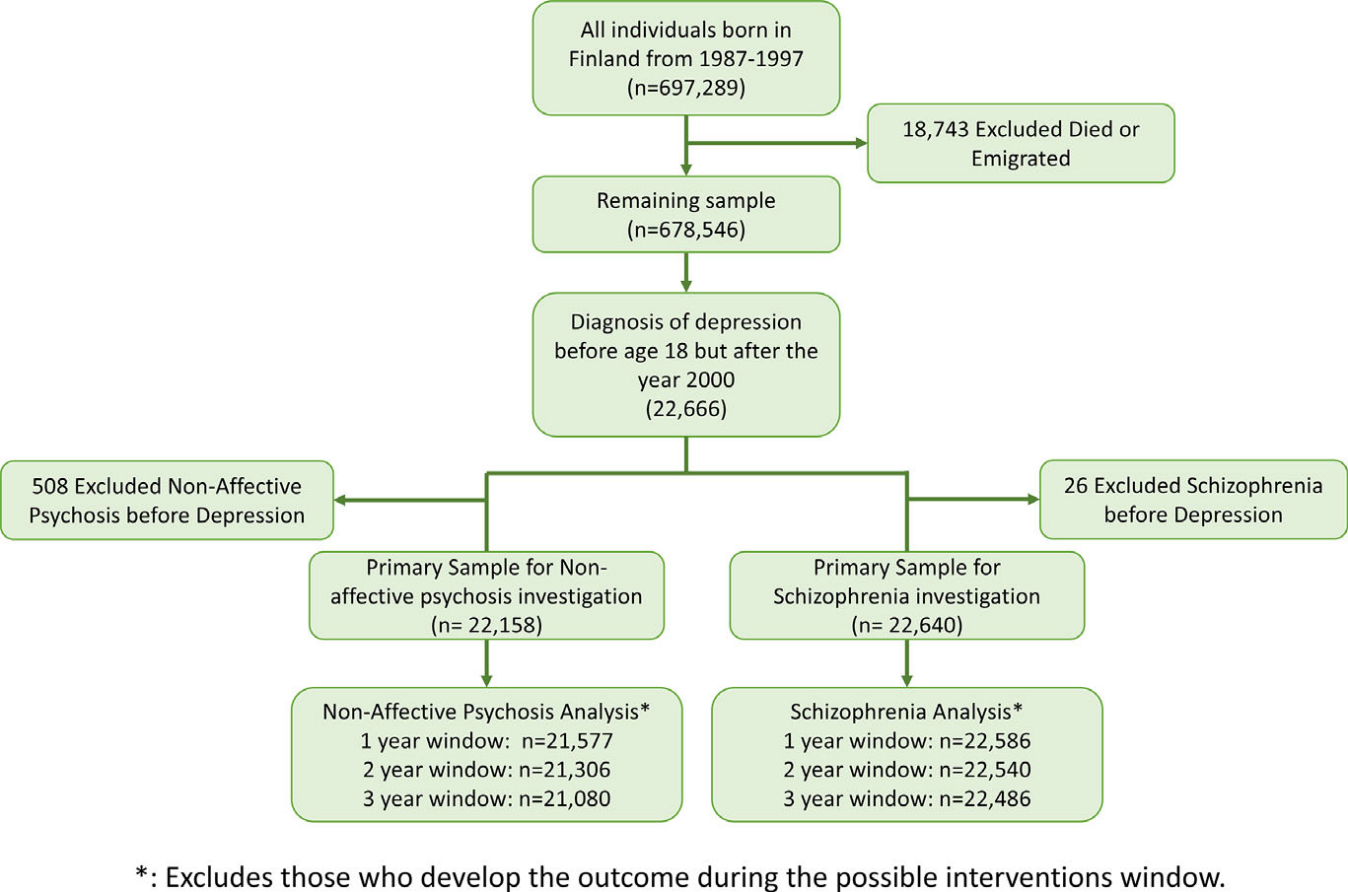
Consort diagram for the investigations.

All individuals born in Finland between 1987 and 1997 were sampled for inclusion using the Medical Birth Registry (see Figure 1). The main samples used for this investigation included individuals diagnosed with depression (primary diagnosis of F32 or F33) in childhood or adolescence (i.e., before age 18 but after January 1, 2000, herein referred to as adolescent depression sample). Finland has a universal healthcare system that accounts for the vast majority of diagnoses and treatment. The care registry for health care only includes data from the public sector. As such, those who only access care through the private sector will not be included in this investigation. A sample of controls demographically matched to the depression sample was also used to investigate the exclusion assumption, which is the assumption that the instrument only affects the outcome through the treatment. Visual description of the sampling can be found in Figure 1.

**Table 1.** Demographic and clinical information in those with and without adolescent depression

Characteristic	Without depression	Adolescent depression (under 18 years)		
		Overall	Depression with SSRI use within 4 years	Depression without SSRI use
*N* (%)	655,880 (96.7)	22,666 (3.3)	13,650 (60.2)	9,016 (39.8)
Female (%)	315,432 (48.1)	16,144 (71.2)	10,351 (75.8)	5,793 (64.3)
Mother’s education (%) Low Intermediate High Missing	120,099 (18.3) 450,316 (68.7) 79,056 (12.0) 6,409 (1.0)	5,971 (26.3) 14,385 (63.5) 2,137 (9.4) 173 (0.8)	3,431 (25.1) 8,806 (64.5) 1,313 (9.6) 100 (0.7)	2,540 (28.2) 5,579 (61.9) 824 (9.1) 73 (0.8)
Father’s education (%) Low Intermediate High Missing	146,047 (22.3) 399,558 (60.9) 97,158 (14.8) 13,117 (2.0)	6,326 (27.9) 13,179 (58.1) 2,597 (11.5) 564 (2.5)	3,710 (27.2) 8,026 (58.8) 1,583 (11.6) 331 (2.4)	2,616 (29.0) 5,153 (57.2) 1,014 (11.3) 233 (2.6)
Parental history of psychosis (%)	–	371 (1.7)	218 (1.6)	153 (1.7)
Parental history of psychiatric inpatient admission (%)	–	3,178 (14.0)	1,877 (13.8)	1,301 (14.4)
Median number of CAP visits prior to diagnosis (IQR)	–	0 (0–1)	0 (0–1)	0 (0–1)
Median number of psychiatric diagnoses before depression (IQR)	–	0 (0–1)	0 (0–1)	0 (0–1)
Inpatient admission prior to depression diagnosis (%)	–	1,027 (4.5)	651 (4.8)	376 (4.2)
Median age of diagnosis (IQR)	–	15.8 (14.5–16.9)	16.0 (14.8–17.1)	15.3 (13.9 –16.6)
Median age of first purchase (IQR)	–	–	16.7 (15.2 – 17.9)	–
Outcome				
Non-affective psychosis (%)	8,366 (1.3)	2,427 (10.7)	1,746 (12.8)	681 (7.6)
Schizophrenia (%)	1,895 (0.3)	457 (2.0)	344 (2.5)	113 (1.3)

Abbreviation: IQR, interquartile range.

a CAP, Child and Adolescent Psychiatric Service.

## Variables

### Demographic and clinical

We report demographic information, including sex at birth, mothers’ and fathers’ education. For individuals with adolescent depression we additionally report the percentage with at least one SSRI reimbursement, parental diagnosis of psychosis prior to the child’s fifth birthday, parental inpatient admission prior to the child’s fifth birthday, the age of first depression diagnosis, the number of child and adolescent psychiatry visits prior to depression diagnosis, the number of other psychiatric diagnoses before depression, an inpatient admission prior to the depression diagnosis, and the age of first prescription.

### Outcome

We report two psychosis outcomes based on ICD-10 diagnostic codes: non-affective psychosis and schizophrenia. Non-affective psychosis included F20.x, F23.x, F28, F29, F22.x, F25.x, and F24. Schizophrenia included F20.x. All psychosis outcomes had to be diagnosed after the depression diagnosis and after the treatment window.

#### SSRI Treatment

The definition of SSRI treatment followed the definition of previous instrumental variable studies examining psychiatric prescriptions with electronic patient records [28, 29]. We identified the cumulative dose of SSRIs prescribed and reimbursed within four possible intervention windows: the first year since depression diagnosis, the first two years since depression diagnosis, the first three years since depression diagnosis, and the first four years since depression diagnosis. Treatment was defined as the cumulative dose of SSRI received within these four treatment windows. These treatment variables were standardized based on a defined daily dose (DDD) per prescription. The DDDs for this investigation were based on the World Health Organization anatomical therapeutic chemical (ATC) code recommendation using code N06ABXX [30]. A list of the individual drug names, ATC codes, the recommended DDDs is provided in Table S1. The treatment variables were standardized such that 0 corresponded to no treatment over the treatment window and 1 corresponded to the cross-SSRI standardized DDD each day for the treatment window. For example, the two-year treatment window cumulative dose was calculated as the standardized sum of the daily DDD in the two-year period (2*365 days). Four years was chosen as the final follow-up as the variability in prescribing preference reduces the further from the original depression diagnosis (see Table S2 and Figure S5).

### Instrumental Variable

The instrument (just-identified case) in each analysis was based on hospital district variability in the prescribing of SSRIs. Finland has a universal healthcare system, and hospital districts are the public administrative regions that provide specialized healthcare services, including psychiatric services. Patients are assigned to a hospital district based on their place of residence. Within this investigation, variability in prescribing of SSRIs across the hospital districts is assumed to be independent of symptom loading (i.e., the assigned treatment center is not determined by severity of depression), conditional on the hospital-level and individual-level confounders listed below.

Four instruments were generated, one for each treatment window. Specifically, hospital district prescriber preference was defined as the average number of DDDs filled for depression among all patients diagnosed with depression in the data, within each hospital district and within the treatment window. A leave-self-out approach was used to ensure each patient’s observation did not contribute to their own likelihood of receiving treatment. Descriptive examination revealed that all hospital districts had more than 30 individuals who received the treatment, ensuring that there were enough individual data points to estimate the mean for each district.

### Confounders

**Figure 2.**
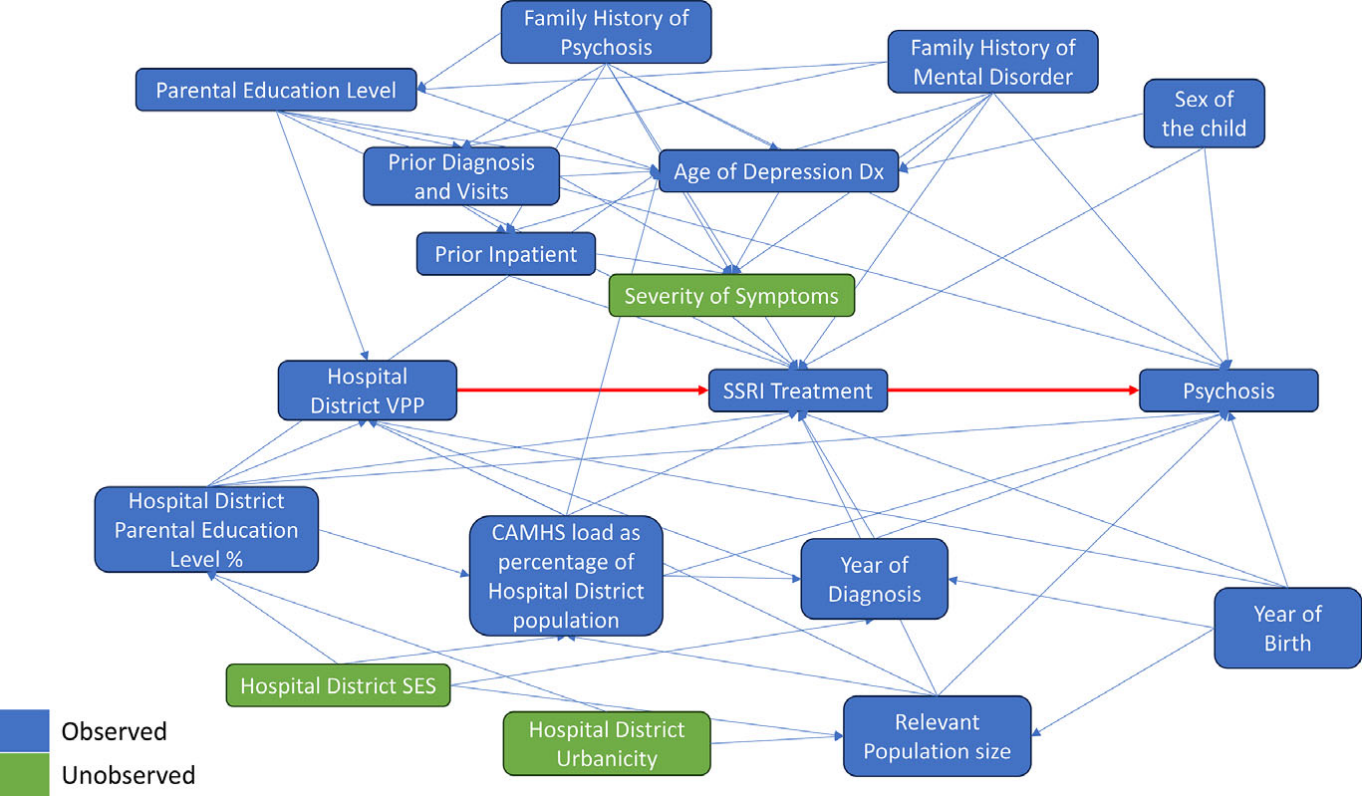
Directed acyclic graph of the relationship between hospital district variability in prescribing practice, SSRI use, and psychosis. The direct path between hospital district VPP (variability in prescribing preference) and SSRI treatment (red) indicates the relevance assumption. A falsification test for the independence assumption is conducted by examining the relationship between each of the observed confounders above with hospital district VPP. A falsification test of exclusion uses a matched sample of controls to examine the relationship between the hospital district VPP for depression and with risk of psychosis. Monotonicity as examined by examining the direction and magnitude of the association between the hospital district VPP and SSRI treatment in the first stage across different levels of the confounders.

A directed acyclic graph was generated for the hypothesized association between SSRI use and psychosis in those with depression (see Figure 2) and used to decide on confounders. These included individual-level variables and hospital district-level variables. The individual-level variables included: parental education (mothers’ and fathers’) at birth, sex at birth, age of diagnosis, the year of the diagnosis and the year of birth, inpatient psychiatry admission before depression diagnosis, number of child and adolescent psychiatry visits prior to depression diagnosis, number of other diagnoses prior to depression, family history of psychosis, and family history of an inpatient stay. The hospital district-level variables included: the population size for each hospital district, the percentage of the population in each hospital district who attended child and adolescent psychiatry services, and the percentage of the population whose mothers’ and fathers’ education level was categorised as high, intermediate, or low, within each hospital district. Educational levels were based on the International Standard Classification of Education 2011 (ISCED) and coded as low (ISCED classes 0 to 2), intermediate (ISCED classes 3–5), and high (ISCED classes 6–8). Missing education level was coded as a separate category.

### Statistical analysis

We report descriptive and clinical characteristics for those with and without depression, as well as descriptive and clinical characteristics when these groups were stratified by SSRI exposure (as a binary or cumulative variable).

We used logistic regression to examine odds ratios for the relationship between depression diagnosis and psychosis (all outcomes). These analyses were conducted in those with and without a depression diagnosis. We examined the relationship between SSRI exposure (as a binary variable and cumulative exposure variables) and psychosis, adjusting for all individual-level and hospital-level confounders. Those who developed the outcome prior to the diagnosis of depression or within the treatment window were excluded from the analyses.

#### Instrumental variable analysis

**Figure 3.**
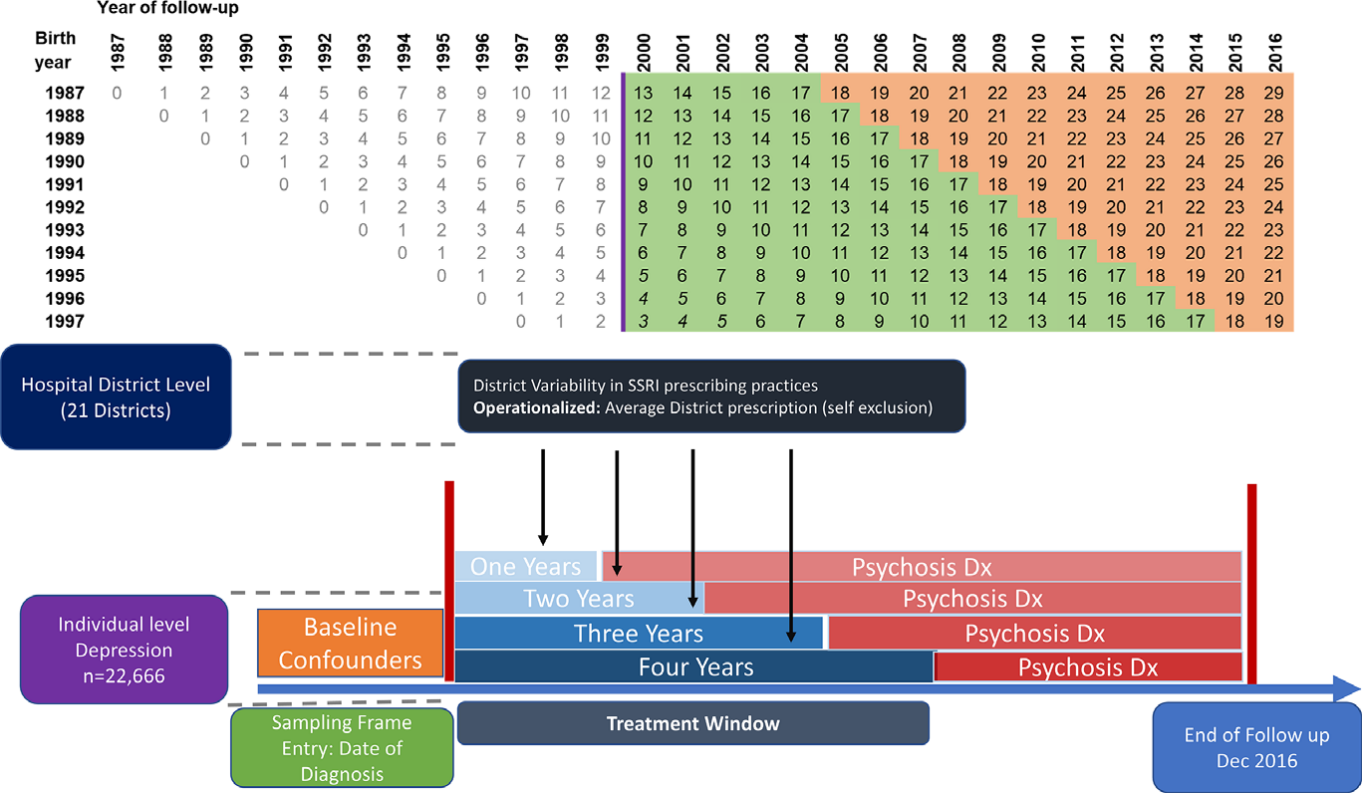
Birth cohort follow-up and schematic of the analysis.

These analyses followed the same approach as Widding-Havneraas and colleagues [28]. A visual description of the procedure can be found in Figure 3. Instrumental variable analysis was conducted in STATA 17 using the ivregress function, which performs a two-stage least squares (2SLS) investigation. Standard errors were clustered at the hospital district level. 2SLS with a binary outcome and a continuous instrument is a linear probability model and produces a treatment effect for those on the margins of treatment. The margins of treatment refer to patients for whom the decision to treat varies from clinician to clinician. Consensus on treatment between clinicians is likely highest for patients with the presence or absence of a clear treatment indicator (for example, with very few symptoms versus a very high number of symptoms). However, consensus between clinicians is likely lower for patients when the indication for treatment is less clear. Specifically, regarding depression, treatment has been observed to vary widely between treatment centers [22]. The coefficient was interpreted as the percentage point change in the probability of the outcome for this group. Analyses were adjusted for the confounders listed in the classical analysis. The estimand is a generalized-local average treatment effect which corresponds to the average causal effect for those on the margins of treatment who range from untreated to treated with the DDD of an SSRI for the entire treatment window.

#### Supplementary Materials. Testable assumptions of IV

There are several assumptions that need to be examined with instrumental variable analyses: relevance, exclusion, independence, monotonicity, and positivity. These were empirically tested through direct or falsification testing (see Supplementary Materials).

### Sensitivity analysis

Three sensitivity analyses were conducted. First, given the known issue of unbounding when interpreting linear probability models with binary outcomes, we additionally used ivprobit to examine the consistency of the results. Second, we examined the association between one-year and two-year variability in prescribing and risk of psychosis across the levels of multiple confounders including: sex (male/female), maternal and paternal education level (low/intermediate/high), age at diagnosis (childhood: before age 13/adolescence: age 13 and above), inpatient admission at any stage during adolescence (no/yes), birth year (median split 1987–1994/1995–1997), and family history of an inpatient psychiatric admission (no/yes). Third, we conducted separate instrumental variable analyses for the most commonly prescribed SSRIs: fluoxetine, citalopram, sertraline, and escitalopram.

## Results

### Descriptive statistics

Our sample included 22,666 individuals (3.3% of the population, 71.1% female) born between 1987 and 1997 who were diagnosed with depression before their 18th birthday. The median age at initial depression diagnosis was 15.8 years (IQR: 14.5–16.9). Those with adolescent depression had lower levels of mothers’ and fathers’ education compared to those without adolescent depression (mother’s education: *X*^2^ = 960.5, p<.001; father’s education: *X*^2^ = 515.6, p<.001).

A higher percentage of females (64.3%) with adolescent depression were dispensed SSRIs compared with males (50.6%, *X*^2^ = 357.3, p<.001). There were no differences between individuals who were dispensed SSRIs and individuals who were not dispensed SSRIs in the proportion of those with a parental history of psychosis (*X*^2^ = 0.5, p = 0.48) or parental history of an inpatient admission (*X*^2^ = 2.4, p = 0.12). Relative to those not dispensed SSRIs, individuals dispensed SSRIs had a marginally higher proportion of inpatient admissions prior to depression (*X*^2^ = 4.4, p = 0.04), number of child and adolescent psychiatry visits (*X*^2^ = 5.2, p = 0.02), and number of diagnoses (*X*^2^ = 5.1, p = 0.02) prior to depression.

### Depression, SSRI use, and psychosis

Relative to those without adolescent depression, individuals with adolescent depression had an elevated risk of subsequent non-affective psychosis (OR: 7.4, CI: 7.05–7.80) and schizophrenia (OR: 5.96, CI: 5.34–6.66).

Of those with adolescent depression, 60.2% were prescribed an SSRI at least once within the first four years after the initial depression diagnosis. In the non-IV model, SSRI use at least once was associated with an increased odds of non-affective psychosis and schizophrenia (see Table 2). Similarly, in the non-IV model, cumulative SSRI use within the first year since diagnosis was associated with an increased risk of non-affective psychosis. Cumulative SSRI exposure within the first two years and first three years since diagnosis was associated with an increased risk of both non-affective psychosis and schizophrenia. These findings, however, are susceptible to confounding, in particular confounding by indication, in that individuals treated with SSRIs are likely to have had more severe depression, and severe depression in youth is more likely to predict later psychotic disorder.

**Table 2.** Odds ratios for the association between SSRI use (binary and cumulative use) with psychosis

	Adolescent depression	
Exposure	Non-affective psychosis OR (95%ile CI)	Schizophrenia OR (95%ile CI)
SSRI use at least once^a^	**2.20** (1.97–2.45)	**2.15** (1.72–2.69)
Cumulative SSRI use		
Within one year since diagnosis^a^	**1.28** (1.13– 1.44)	1.16 (0.87–1.54)
Within two years since diagnosis^a^	**1.56** (1.35–1.81)	**1.48** (1.06–2.07)
Within three years since diagnosis^a^	**1.83** (1.54– 2.19)	**1.71** (1.14– 2.56)

a Analysis adjusted for participants’ birth year, age of first diagnosis, sex, mothers’ and fathers’ education levels, year of diagnosis, the hospital district-level, the hospital district population size for the given cohort, and the percentage of each education level within the hospital district. Emboldened values denote significant associations at p < .05.

### Instrumental variable analysis

There was notable variability in prescribing practice for SSRIs across hospital districts for the first three years of SSRI treatment for adolescent depression, supporting its validity as an IV (see Table 3, Table S2, Table S8, Figure S1, and Figure S5). By the fourth year, the variability in prescribing had notably reduced (see Table S2, Figure S5). Thus, we did not conduct an investigation into variability in prescribing for the four-year treatment window and risk of psychosis. The results of the first stage of the analyses for the remaining three instruments indicated that variability in prescribing was associated with treatment (F-statistic range: 187.8–383.3, see Table 3). The instrumental variable analyses indicated that sustained treatment with SSRIs for one year, two years, or three years had no relationship with either non-affective psychosis or schizophrenia (Table 3).

**Table 3.** Instrumental variable analysis results of the relationship between variability in prescribing and risk of psychosis

Intervention windows	Adolescent depression	
	Non-affective psychosis	Schizophrenia
One-year		
Number of outcomes	1,338	377
F-statistic (IV relevance)	303.8	342.2
IV effect 2SLS B (95%ile CI)	0.04 (−0.01 to 0.09)	0.03 (−0.01 to 0.06)
Two-years		
Number of outcomes	1,067	331
F-statistic (IV relevance)	266.4	336.2
IV effect 2SLS B (95%ile CI)	0.02 (−0.06 to 0.09)	0.02 (−0.02 to 0.06)
Three-years		
Number of outcomes	841	277
F-statistic (IV relevance)	180.2	252.6
IV effect 2SLS B (95%ile CI)	−0.02 (−0.08 to 0.05)	0.01 (−0.03 to 0.05)

*Note*: Analysis conducted using ivregress 2sls with clustering at the hospital district level. Those who develop the outcome during the treatment window were removed from the analysis.

### Supplementary analysis and results

A full description of the results of the sensitivity analyses is provided in Supplementary Materials. Briefly, first, analysis using a probit instrumental variable model replicated the findings from the main analysis (see Table S3). Second, we investigated the empirically testable assumptions of instrumental variable analysis. These include a direct testing of the instrument’s relevance and falsification tests of the exclusion, independence, and monotonicity assumptions. The results of the directly tested assumptions and the falsifiable tested assumptions suggest that our estimates are valid for causal interpretation.

Third, we found no association between SSRI treatment and subsequent psychosis within any strata of multiple demographic and clinical confounders, including sex, maternal education, age of diagnosis, adolescent inpatient admission, birth year, and family history of an inpatient admission (see Tables S5). Finally, none of the individual SSRI medications was associated with a reduced risk of psychosis (see Table S6 and Table S7).

## Discussion

Several studies have highlighted that adolescent depressive disorders frequently precede psychotic disorders [6, 31, 32], and researchers have hypothesized that SSRI treatment may potentially reduce the risk of psychosis [9, 10]. Using national healthcare registry linkage data, we identified all young people born in Finland from 1987 to 1997 who were diagnosed with a depressive disorder in child and adolescent psychiatry services and followed them into adulthood, up to age 30 years. We applied quasi-experimental methods to assess for a potential causal relationship between SSRI treatment and subsequent psychosis risk. Contrary to our expectations, we found no evidence to support the idea that SSRI treatment of depression in adolescence reduces the risk of later psychotic disorder.

The advantage of using hospital district variation in prescribing as an instrumental variable is that unobserved confounding (such as confounding by indication) can be accounted for by exploiting conditional as-if-random variability in treatment. This allows for the estimation of a causal effect provided certain assumptions are met. Some of these assumptions are directly testable (relevance) while others are examined by falsification testing or defended on a theoretical basis (exclusion and independence) [23, 33, 34].

Relevance was directly tested based on the strength of the association between the instrument and SSRI treatment. We observed that variability in SSRI prescribing for depression was strongly associated with the cumulative amount of SSRI treatment received. Exclusion, the assumption that the instrument only affects the outcome through the treatment, was indirectly tested using a matched sample of controls. We observed that hospital district-level variability in prescribing SSRIs for depression was *not* associated with psychosis in individuals not exposed to SSRIs.

Lastly, we indirectly tested the independence assumption, which assumes that the instrument is independent of unmeasured confounding. This was tested by examining the relationship between the measured confounders and the instrument. We observed no association between the confounders and variability in SSRI prescribing for depression. Whilst we cannot completely exclude the possibility of unmeasured confounding, we found little to no evidence to suggest that confounding was occurring. Together, these results showed that, in keeping with other studies [16, 21, 29, 34], variability in SSRI prescribing between hospital districts met the assumptions to be considered a valid instrument in the current study.

Our findings raise questions about the “intervention as prevention” hypothesis in mental health. This relates to the widespread assumption that treatment of common mental health disorders in youth may prevent the development of severe mental illnesses in adulthood [12]. To date, only a small number of studies have formally tested this hypothesis, with conflicting results [35–37]. Our findings, while specific to the long-term (lack of) effect of depression treatment on subsequent psychosis risk, also demonstrate, more broadly, that it would be a mistake to assume that treatments of common mental disorders in childhood reduce the risk of severe mental illness in adulthood. In that regard, our results highlight the need for far more robust research to assess the effect of youth mental health interventions on long-term outcomes.

Our findings also point to the need for new precision approaches to the prevention of severe mental illness (SMI), not simply based on the assumption that treatment of common mental health problems in youth will reduce SMI risk. This might include research looking at factors that directly impact mechanisms believed to underlie the development of severe mental illness. From a biological perspective, this might include assessing the effects of medications that impact, for example, neuroinflammation and synaptic pruning, including anti-inflammatory medications [38, 39], glucagon-like peptide-1 agonists [40–42], and medications affecting the complement system [43, 44]. This would similarly include research on psychosocial candidates implicated in the etiology of SMI, involving robust causal assessments of the effects of interventions designed, for example, to reduce childhood deprivation and maltreatment [45], parenting interventions [46, 47], and social inclusion programs [48].

Strengths of the present study include the use of a quasi-experimental design, which allowed us to estimate causal associations and overcome the issue of confounding-by-indication that affects typical observational studies. We extensively assessed the instrumental variable assumptions and conducted sensitivity analyses, including confounder stratification and individual SSRI analyses. These allowed us to show that the findings are broadly consistent across demographic subgroups and for the most commonly prescribed SSRIs. Further strengths include the use of a total population prospective birth cohort, administrative data that avoids recall or interviewer biases, and long-term follow-ups, which are not typically possible with RCT designs. While we can state that individuals using SSRIs were prescribed and reimbursed for monthly treatments, we cannot categorically confirm that individuals adhered to the treatment regimen. However, we investigated sustained treatment over time, and it is likely that individuals who were reimbursed on multiple occasions were more likely to have taken a greater quantity of the treatment than those who obtained fewer reimbursements.

In conclusion, using robust quasi-experimental methods, we did not find evidence that SSRI treatment of adolescent depressive disorders was associated with a subsequent reduced risk of psychotic disorders. These findings raise important questions for the “intervention as prevention” hypothesis that treatment of mental disorders in youth reduces the risk of later severe mental illness. Our findings highlight the need for more methodologically rigorous research in this area and more robust studies on the long-term effects of youth mental health treatments, including any putative preventative effects.

## Supporting information

10.1192/j.eurpsy.2025.10050.sm001 Healy et al. supplementary material Healy et al. supplementary material

## Data Availability

Access to the data was provided through the Finnish Institute for Health and Welfare, Finland. As data processors, rather than controllers, we do not have permission to provide public access to the data.

## Supplementary material

The supplementary material for this article can be found at http://doi.org/10.1192/j.eurpsy.2025.10050.
