# Does treatment of adolescent depression reduce risk of later psychosis: A quasi-experimental study of selective serotonin reuptake inhibitor treatment in a total population cohort – CORRIGENDUM

**DOI:** 10.1192/j.eurpsy.2025.10079

**Published:** 2025-09-23

**Authors:** Colm Healy, Kirstie O’Hare, Ulla Lång, Martta Kerkelä, Jonah Byrne, Juha Veijola, Anna Pulakka, Johanna Metsälä, Eero Kajantie, Ian Kelleher

**Keywords:** depression, prevention, psychosis, schizophrenia, SSRIs, emulated target trial, corrigendum

This article was originally published with a spelling error in the name of author Kirstie O’Hare. This has now been rectified and this corrigendum published.
