# Supplementary material for: Does treatment of adolescent depression reduce risk of later psychosis: A quasi-experimental study of selective serotonin reuptake inhibitor treatment in a total population cohort
Source: Eur Psychiatry. 2025 Jun 25;68(1):e82. doi: 10.1192/j.eurpsy.2025.10050 (PMC12188345; doi:10.1192/j.eurpsy.2025.10050)

**Supplementary Materials**

**Table of Content.**

| **Content** | **Page** |
| --- | --- |
| **Supplementary Methods and Results.** Testing the assumptions of instrumental variable | 3 |
| **Supplementary Results. *SSRI stratified analysis.*** | 5 |
| **Table S1.** The recommended defined daily dose for each SSRI based on the WHO ATC recommendation. | 6 |
| **Table S2.** Variability in Prescribing practice based on the standardized follow up daily defined dose across the hospital districts. | 7 |
| **Table S3.** Instrumental variable analysis results of the relationship between variability in prescribing and risk of psychosis from ivprobit models with reported marginal effects. | 8 |
| **Table S4.** Beta and CI examining the relationship between variability in prescribing (reduced form) and psychosis in the matched control population. | 9 |
| **Table S5.** The mean variability in hospital prescribing and association between non-affective psychosis across different strata of the confounders in those with adolescent depression. | 10 |
| **Table S6.** Descriptive Statistics, variability in prescribing and odd of non-affective psychosis for each SSRI within those who have adolescent depression. | 12 |
| **Table S7.** The results of the instrumental variable analysis when stratified by type of treatment. | 13 |
| **Table S8.** The median prescribing preference for each hospital district in each year. | 14 |
| **Figure S1.** Balance plots displaying the association between the instruments and confounders with cumulative SSRI use within one-, two- and three-year treatment window in those with adolescent depression. | 15 |
| **Figure S2.** Balance plots displaying the association between the confounders and the instrument use for the one-, two- and three-year treatment window in those with adolescent depression. | 16 |
| **Figures S3.**  Balance plots displaying the standardised percentage bias across confounders in the matched control sample to those with adolescent depression. | 17 |
| **Figures S4.** Balance plots displaying the association between the confounders and the instrument use for the one- and two-year treatment window in those with adolescent depression. | 18 |
| **Figures S5.** The hospital district variability in prescribing for those with depression across each of the years as well as medication trajectories stratified into tertiles. | 19 |

**Supplementary Methods and Results. *Testing the assumptions of instrumental variable***

There are several assumptions that need to be examine with instrumental variable analysis: relevance, exclusion, independence, monotonicity, positivity. These were directly or indirectly empirically tested where possible.

**Method.** Relevance indicated the strength of the instrument. This is statistically tested based on F-statistic from the first stage of the model. Exclusion assumes that the effect of prescriber preference in individuals with a given diagnoses must affect the outcome through the treatment with SSRI’s and not through other methods. This is not directly testable. A falsification test was conducting using a matched sample of controls (general population without adolescent depression and adolescent psychiatric service use) to examine for association between the average treatment effect in the hospital district (reduced form) and risk of psychosis. This analysis used linear regression with standard errors clustered by birth hospital district. Independence assumes that the instrument is unconfounded, that is to say unrelated to all confounders. This is not directly testable due to unmeasured confounding, but a falsification test can be conducted. This test examined the relationship between the measure confounders with the variability in hospital prescribing practices both jointly and independently. We report the joint F-statistic and individual associations from a multivariable linear regression model in which variability in prescribing preference was the outcome and the measured confounders were the exposures. Each confounder was included, and standard errors were clustered at the hospital district level. Monotonicity assumes that the instrument affects the treatment in only one direction for each individual. This cannot be empirically tested but was indirectly assess by examining the direction and the magnitude of the relationship between variability in provider preference with treatment across multiple levels of confounders. Confounders examined included: sex (male/female), maternal and paternal education level (low/intermediate/high), age at diagnosis (childhood: before age 13/adolescence: age 13 and above), inpatient admission at any stage during adolescence (no/yes) and birth year (median split 1987-1994/1995-1997) and family history of if an inpatient psychiatric admission (no/yes). For each level of the confounders, we report the F statistic and the coefficient beta for the first stage of the instrumental variable analysis. Positivity assumes that across the levels of confounders at least some patients received a non-zero value for provider preference. This was assessed by descriptively examining provider preference across levels of the confounders.

***Relevance:*** In both samples the F-statistics indicate that there was a very strong relationship between variability in prescribing practices and the cumulative daily dose of SSRI use over the treatment period (partial F-statistic range: 180.2 – 342.2). This surpasses the traditional estimate required to meet the relevance assumption (F>10, see Staiger & Stock, 1997), the more conservative estimate (F>50, see Keane & Neal,2024) and is also beyond the t-ratio adjustment based on the tF critical value function (F >104) which produces an even more conversative standard error estimates (Lee et al., 2022). Moreover, comparing the relative associations between the confounders with treatment and instrument with the treatment suggests that hospital district variability in prescribing has a notably stronger relationship with treatment than any confounder (see Figure S1).

***Exclusion:*** A general population sample matched at a 5:1 ratio to those with depression for year of birth, sex parental education and hospital district at birth (see Figure S3). There was strong agreement (85.5%) between hospital district at birth and hospital district at diagnosis for those with depression (kappa=0.83). The reduced form analysis in the control sample revealed that there was no association between hospital district prescribing practice variability and psychosis (see Table S4). Given these individuals were not exposed to SSRIs it suggests that any change in the risk of psychosis, in those exposed to SSRIs is likely a result of their exposure.

***Independence:*** Results for each of our intervention windows indicated that there was no association between the hospital district variability in prescribing and the observed confounders. (see Figure S2). The joint F statistic, including all confounders, indicated a notably weak combined association with the instrument in spite of the number of measured confounders; year one F = 10.7; year two F = 19.9; and year three F = 23.2.

***Monotonicity:*** Across each level of each confounder there was a positive linear relationship between the hospital district variability in prescribing and the cumulative SSRI exposure (see Table S5). The effect size of the relationship between the variability in prescribing and cumulative SSRI exposure overlapped across almost all levels of each confounder. There was some variability in the F statistic size with a lower F noted in confounder levels with smaller group sizes. The only circumstance where there was a classically weak F statistic (F < 10) and a non-significant relationship between the instrument with treatment was for those with mothers with high levels of education by the birth of their child. This group were notably small in size then the other two levels (low and intermediate) and the first stage beta coefficient remained in the same direction as the other levels.

**Positivity.** Results indicate that across all strata of the confounders there were non-zero estimates for the variability in prescribing. That is to say, there was a non-zero probability of receiving treatment (see Table S5).

**References**

Staiger, D. O., & Stock, J. H. (1997). Instrumental variables regression with weak instruments. *Econometrica* 65 (3): 557–86

Keane, M. P., & Neal, T. (2024). A practical guide to weak instruments. *Annual Review of Economics*, *16*. 185-212

Lee, D. S., McCrary, J., Moreira, M. J., & Porter, J. (2022). Valid t-ratio Inference for IV. *American Economic Review*, *112*(10), 3260-3290. **Results. *SSRI stratified analysis.*** We investigated the SSRIs which accounted for the majority of the prescribed SSRIs; fluoxetine, citalopram, sertraline and escitalopram. The results of each investigation are reported in the materials (see Table S6). Fluoxetine was the most commonly reported medication and was prescribed at least once in 32% of cases of depression. In the classic analysis, those prescribed with each medication within the four years since diagnosis were associated with an increased risk of psychosis relative to those without that prescription. Cumulative use of fluoxetine, sertraline and escitalopram were associated with an increased risk of psychosis in those with depression, however there is likely unmeasured confounding, and these were also the most prescribed SSRI.

Descriptives statistics indicated that the median prescribing for each SSRI and within each intervention window was low (zero reflects no treatment and one reflects the standardised DDD each day for the treatment window., see Table S6). This was particularly the case for citalopram, sertraline and escitalopram. Variability in prescribing, as assessed by the 10^th^ to 90^th^ percentile confidence intervals, was also particularly limited for citalopram. Variability in prescribing was strongly related to the cumulative SSRI use within the treatment windows (F-statistic range: 50.7-5836.2, see Table S7). Fluoxetine, sertraline and escitalopram were not associated with risk of psychosis in those with adolescent depression. Balance plots suggested that the instrument was independent of measured confounding (see Figure S4).

**Table S1.** The recommended defined daily dose for each SSRI based on the WHO ATC recommendation.

| SSRI Name | ATC code | DDD ATC-recommended |
| --- | --- | --- |
| Fluoxetine | N06AB03 | 20mg |
| Citalopram | N06AB04 | 20mg |
| Paroxetine | N06AB05 | 20mg |
| Sertraline | N06AB06 | 50mg |
| Fluvoxamine | N06AB08 | 100mg |
| Escitalopram | N06AB10 | 10mg |

Note: ATC is the Anatomical Therapeutic Chemical code; and DDD is the defined daily dose.

**Table S2.** Variability in Prescribing practice based on the standardized follow up daily defined dose across the hospital districts.

| **Years follow up from Diagnosis** | **Adolescent Depression** | | |  |
| --- | --- | --- | --- | --- |
|  | **Median** | **10%ile** | **90%ile** | **Diff** |
| One year | 0.29 | 0.19 | 0.37 | 0.18 |
| Two years | 0.25 | 0.18 | 0.32 | 0.14 |
| Three years | 0.23 | 0.17 | 0.28 | 0.11 |
| Four years | 0.21 | 0.17 | 0.24 | 0.07 |

**Table S3.** Instrumental variable analysis results of the relationship between variability in prescribing and risk of psychosis from ivprobit models with reported marginal effects.

| **Different IVs** | **Adolescent Depression** | |
| --- | --- | --- |
|  | **Non-Affective Psychosis** | **Schizophrenia** |
| **One-year variability in prescribing** |  |  |
| Number of Outcomes | 1,338 | 377 |
| IV effect probit model (95%ile CI) | 0.03  (-0.01 to 0.08) | 0.03  (-0.01 to 0.06) |
| **Two-year variability in prescribing.** |  |  |
| Number of Outcomes | 1,067 | 331 |
| IV effect probit model (95%ile CI) | 0.02  (-0.07 to 0.10) | 0.03  (-0.01 to 0.06) |
| **Three-year variability in prescribing.** |  |  |
| Number of Outcomes | 841 | 277 |
| IV effect probit model (95%ile CI) | -0.02  (-0.13 to 0.09) | 0.02  (-0.02 to 0.06) |

Note. Analysis conducted using ivprobit with clustering at the hospital district level. Those who develop the outcome during the treatment window were removed from the analysis

**Table S4.** Beta and CI examining the relationship between variability in prescribing (reduced form) and psychosis in the matched control population.

|  | **Matched Control Population** | |
| --- | --- | --- |
| **Years of Follow up** | **Non-Affective Psychosis** | **Schizophrenia** |
| One year – IV effect 2SLS Beta (95%ile CI) | -0.01  (-0.02 to 0.02) | -0.00  (-0.01 to 0.01) |
| Two year – IV effect 2SLS Beta (95%ile CI) | 0.01  (-0.03 to 0.04) | -0.00  (-0.02 to 0.01) |
| Three year – IV effect 2SLS Beta (95%ile CI) | 0.01  (-0.03 to 0.05) | -0.01  (-0.02 to 0.01) |

**Table S5.** The mean variability in hospital prescribing and association between non-affective psychosis across different strata of the confounders in those with adolescent depression.

|  | **One-year Variability.** | | |  | **Two-year Variability** | | |  |
| --- | --- | --- | --- | --- | --- | --- | --- | --- |
| **Confounder strata** | **Median**  **(10ile-90ile)** | **First stage (F-statistic)** | **First stage Beta** | **IV Beta (95%ile CI)** | **Median**  **(10ile-90ile)** | **First stage (F- statistic)** | **First stage Beta** | **IV Beta (95%ile CI)** |
| Sex |  |  |  |  |  |  |  |  |
| Male (n = 6,522) | 0.23  (0.17-0.32) | 36.3 | 0.61  (0.40-0.82) | 0.13  (-0.04 to 0.29) | 0.19  (0.16-0.26) | 31.9 | 0.55  (0.35-0.76) | 0.06  (-0.13 to 0.26) |
| Female (n=16,144) | 0.32  (0.20-0.40) | 334.6 | 0.83  (0.74-0.93) | -0.03  (-0.09 to 0.04) | 0.28  (0.19-0.33) | 297.5 | 0.84  (0.75-0.94) | -0.04  (-0.12 to 0.05) |
| Mothers Education Level |  |  |  |  |  |  |  |  |
| Low (n = 5,971) | 0.25  (0.18-0.30) | 18.9 | 0.62  (0.34-0.90) | 0.08  (-0.12 to 0.28) | 0.21  (0.16-0.25) | 19.6 | 0.62  (0.34-0.90) | 0.07  (-0.23 to 0.36) |
| Intermediate (n = 14,385) | 0.31  (0.19-0.39) | 318.2 | 0.80  (0.71-0.88) | 0.03  (-0.04 to 0.09) | 0.27  (0.18-0.33) | 297.6 | 0.77  (0.69-0.87) | 0.03  (-0.08 to 0.13) |
| High (n = 2,137) | 0.32  (0.22-0.41) | 3.8 | 0.32  (0.01-0.65) | 0.29  (-0.21 to 0.80) | 0.29  (0.22-0.36) | 2.8 | 0.29  (-0.05-0.64) | 0.26  (-0.30 to 0.81) |
| Age of Diagnosis |  |  |  |  |  |  |  |  |
| Childhood (under 13, n = 2,229) | 0.08  (0.02-0.16) | 30.1 | 0.65  (0.42-0.88) | 0.02  (-0.27 to 0.31) | 0.08  (0.05-0.16) | 39.2 | 0.64  (0.44-0.84) | 0.05  (-0.32 to 0.42) |
| Adolescence (13 and over n =20,437) | 0.31  (0.20-0.39) | 405.3 | 0.84  (0.76-0.93) | 0.04  (-0.01 to 0.09) | 0.27  (0.19-0.33) | 356.8 | 0.84  (0.75-0.93) | 0.02  (-0.07 to 0.11) |
| Adolescent Inpatient Admission |  |  |  |  |  |  |  |  |
| No (n = 14,003) | 0.25  (0.19-0.34) | 272.0 | 0.75  (0.66-0.84) | 0.00  (-0.04 to 0.04) | 0.20  (0.16-0.26) | 234.1 | 0.72  (0.62-0.80) | -0.03  (-0.07 to 0.01) |
| Yes (n = 8,663) | 0.33  (0.23-0.41) | 166.5 | 0.81  (0.69-0.93) | -0.03  (-0.11 to 0.05) | 0.29  (0.22-0.39) | 122.7 | 0.80  (0.66-0.95) | 0.03  (-0.08 to 0.13) |
| Birth Year |  |  |  |  |  |  |  |  |
| 1987-1994 (n = 15,066) | 0.27  (0.17-0.33) | 161.6 | 0.81  (0.68-0.93) | 0.07  (0.01 to 0.14) | 0.23  (0.17-0.28) | 123.3 | 0.77  (0.64-0.91) | 0.04  (-0.05 to 0.12) |
| 1995-1997 (n = 7,600) | 0.33  (0.23-0.41) | 173.6 | 0.71  (0.61-0.82) | -0.03  (-0.12 to 0.05) | 0.29  (0.21-0.37) | 122.3 | 0.74  (0.61-0.87) | 0.03  (-0.08 to 0.13) |
| Family History of Inpatient Admission |  |  |  |  |  |  |  |  |
| No (n = 19,488) | 0.30  (0.18-0.37) | 227.2 | 0.78  (0.68-0.88) | 0.03  (-0.02 to 0.07) | 0.25  (0.18-0.31) | 185.8 | 0.77  (0.66-0.88) | 0.00  (-0.08 to 0.07) |
| Yes (n = 3,178) | 0.26  (0.18-0.37) | 57.9 | 0.65  (0.48-0.82) | 0.09  (-0.12 to 0.30) | 0.23  (0.17-0.33) | 41.1 | 0.61  (0.42-0.80) | 0.12  (-0.18 to 0.43) |

**Table S6.** Descriptive Statistics, variability in prescribing and odd of non-affective psychosis for each SSRI within those who have adolescent depression.

|  | **Adolescent Depression** | | | |
| --- | --- | --- | --- | --- |
| **Variables** | **Fluoxetine**  **N06AB03** | **Citalopram**  **N06AB04** | **Sertraline**  **N06AB06** | **Escitalopram**  **N06AB10** |
| **Proportion Prescribed % (n)** | 32.2 (7,294) | 11.5 (2,595) | 14.4 (3,274) | 22.4 (5,067) |
| **Female % (n)** | 76.8 (5,603) | 75.7 (1,965) | 78.0 (2,555) | 77.7 (3,937) |
| **Median Age of First Prescribed (IQR)** | 16.3  (15.1 – 17.5) | 18.5  (16.3 – 21.0) | 18.1  (16.4 – 21.7) | 18.2  (16.7 – 21.5) |
| **Defined daily dose of each SSRI** |  |  |  |  |
| One year Median (10-90%) | 0.12  (0.06-0.16) | 0.02  (0.02-0.04) | 0.05  (0.03-0.08) | 0.06  (0.04-0.11) |
| Two years Median (10-90%) | 0.11  (0.05-0.13) | 0.02  (0.02-0.04) | 0.05  (0.02-0.07) | 0.05  (0.04-0.10) |
| **Non-affective Psychosis (%)** | 10.4% | 10.4% | 8.9% | 8.8% |
| SSRI use at least once^a^ | **1.51**  (1.36 – 1.67) | **1.33**  (1.16 – 1.55) | **1.46**  (1.27 – 1.68) | **1.50**  (1.33 – 1.69) |
| Cumulative SSRI use within 1 year since diagnosis^a^ | **1.21**  (1.01 – 1.44) | 1.22  (0.86 – 1.72) | 1.05  (0.84 – 1.34) | **1.38**  (1.11 – 1.71) |
| Cumulative SSRI use within 2 year since diagnosis^a^ | **1.46**  (1.20 – 1.78) | 0.98  (0.61 – 1.60) | **1.32**  (1.01 – 1.72) | **1.82**  (1.41 – 2.36) |

Note: IQR is the interquartile range.

**Table S7.** The results of the instrumental variable analysis when stratified by type of treatment.

|  | **Adolescent Depression** | | | |
| --- | --- | --- | --- | --- |
| **Different IVs** | **Fluoxetine**  **N06AB03** | **Citalopram**  **N06AB04** | **Sertraline**  **N06AB06** | **Escitalopram**  **N06AB10** |
| **One-year VPP** |  |  |  |  |
| Number of Outcomes | 1338 | 1338 | 1338 | 1338 |
| F-statistic (IV relevance) | 280.7 | 80.6 | 5209.9 | 563.8 |
| IV effect 2SLS (95%ile CI) | 0.03  (-0.08 to 0.13) | 0.21  (0.02 to 0.41) | 0.01  (-0.06 to 0.08) | 0.05  (-0.07 to 0.17) |
| **Two-year VPP.** |  |  |  |  |
| Number of Outcomes | 1,067 | 1,067 | 1,067 | 1,067 |
| F-statistic (IV relevance) | 167.1 | 50.7 | 5836.2 | 294.3 |
| IV effect 2SLS (95%ile CI) | -0.04  (-0.15 to 0.08) | 0.22  (-0.03 to 0.48) | 0.01  (-0.09 to 0.10) | 0.04  (-0.09 to 0.18) |

**Table S8.** Median Prescribing across the hospital districts.

| **Hospital Districts** | **Median Year 1** | **Median Year 2** | **Median Year 3** | **Median Year 4** |
| --- | --- | --- | --- | --- |
| Southwest Finland | 0.28 | 0.23 | 0.21 | 0.19 |
| Satakunta | 0.37 | 0.33 | 0.29 | 0.26 |
| Kanta-Häme | 0.18 | 0.18 | 0.17 | 0.16 |
| Pirkanmaa | 0.23 | 0.22 | 0.20 | 0.19 |
| Åland | 0.47 | 0.38 | 0.37 | 0.35 |
| Päijät-Häme | 0.33 | 0.29 | 0.26 | 0.24 |
| Kymenlaakso | 0.36 | 0.29 | 0.25 | 0.22 |
| South Karelia | 0.25 | 0.21 | 0.19 | 0.17 |
| South Savo | 0.36 | 0.31 | 0.27 | 0.24 |
| Itä-Savo | 0.25 | 0.19 | 0.16 | 0.15 |
| North Karelia | 0.40 | 0.33 | 0.29 | 0.27 |
| North Savo | 0.24 | 0.20 | 0.18 | 0.17 |
| Central Finland | 0.38 | 0.32 | 0.28 | 0.24 |
| South Ostrobothnia | 0.37 | 0.30 | 0.27 | 0.23 |
| Vaasa | 0.28 | 0.25 | 0.23 | 0.20 |
| Central Ostrobothnia | 0.24 | 0.22 | 0.20 | 0.18 |
| North Ostrobothnia | 0.17 | 0.16 | 0.16 | 0.15 |
| Kainuu | 0.33 | 0.28 | 0.25 | 0.23 |
| Länsi-Pohja | 0.16 | 0.12 | 0.10 | 0.09 |
| Lappi | 0.26 | 0.22 | 0.20 | 0.18 |
| Helsinki and Uusimaa | 0.29 | 0.25 | 0.23 | 0.21 |

**Figure S1.** Balance plots displaying the association between the instruments and confounders with cumulative SSRI use within one-, two- and three-year treatment window in those with adolescent depression.

**
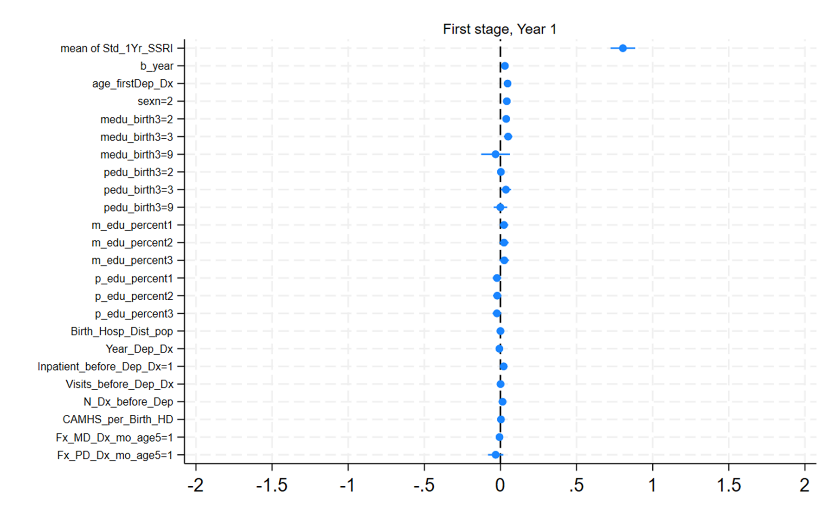

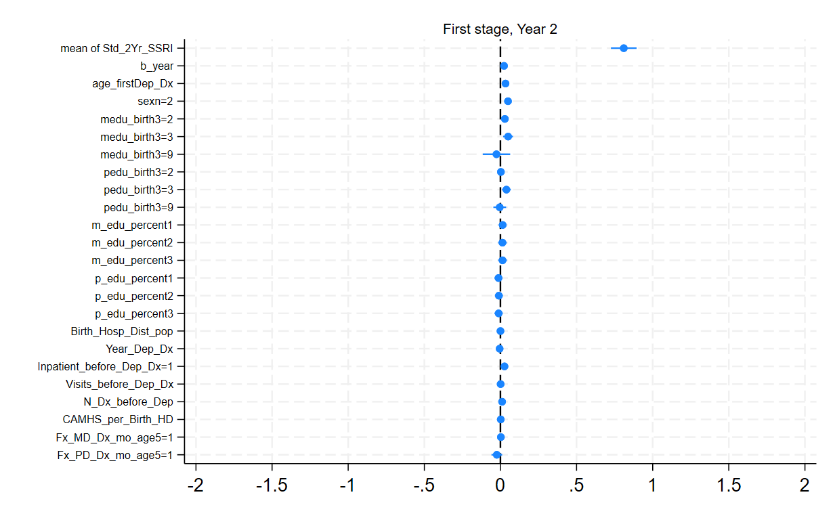
**

**
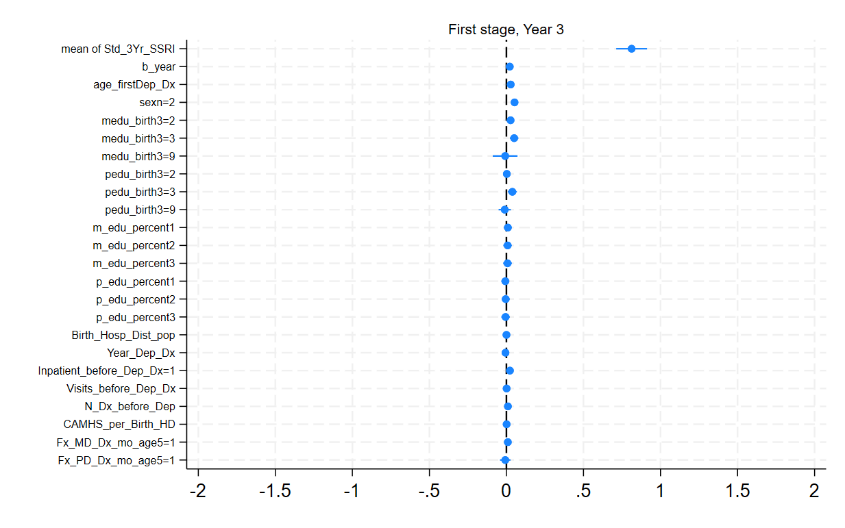
**

**Figure S2.** Balance plots displaying the association between the confounders and the instrument use for the one-, two- and three-year treatment window in those with adolescent depression.

**
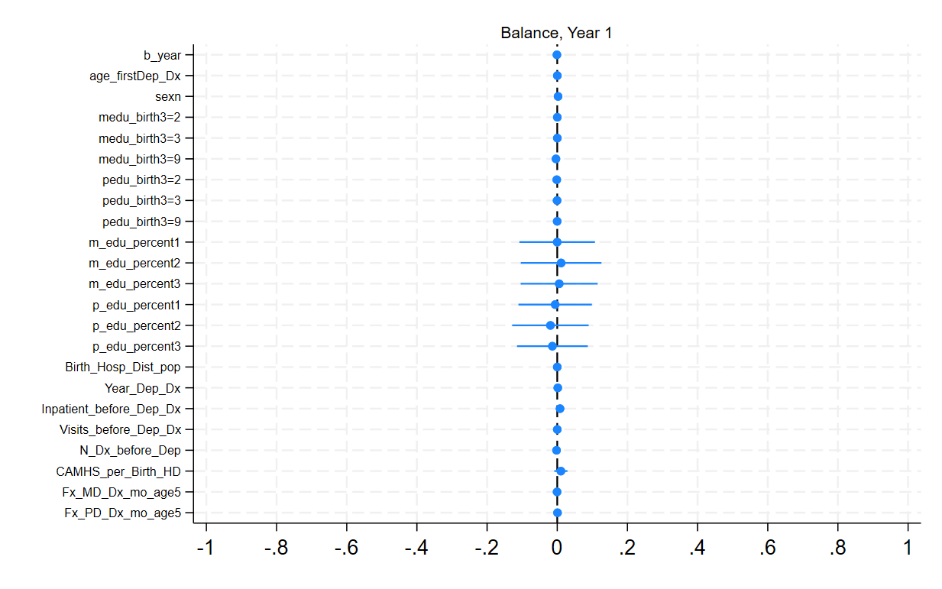

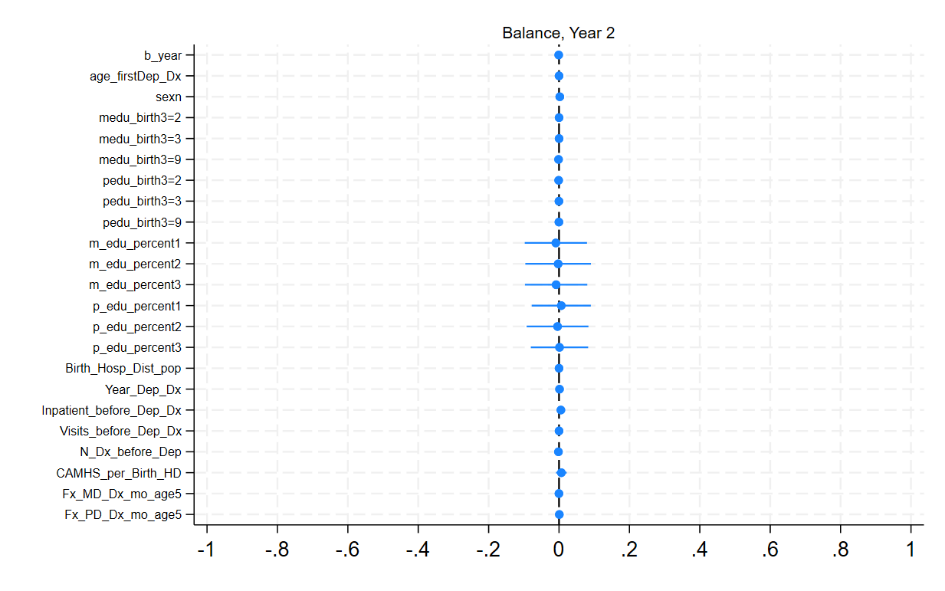
**

**
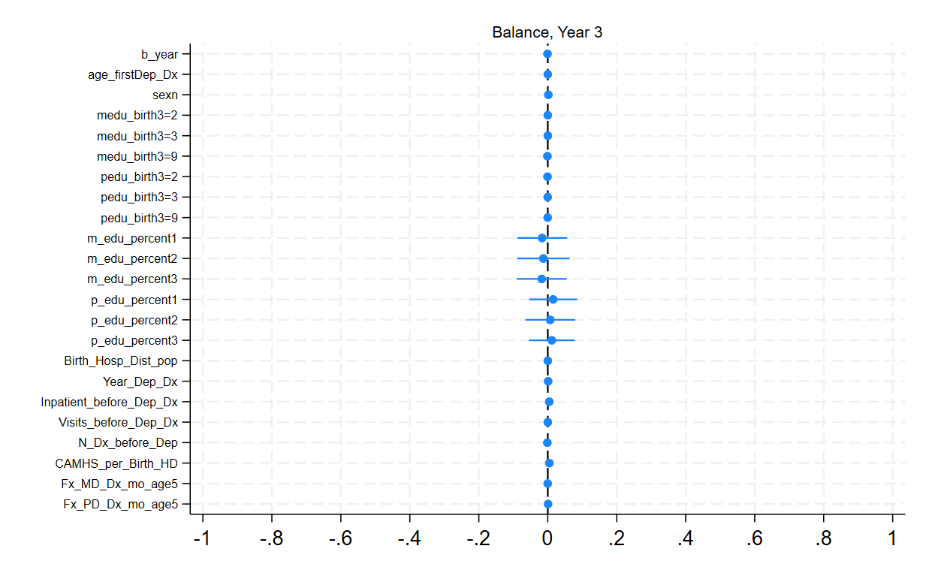
**

**Figures S3.**  Balance plots displaying the standardised percentage bias across covariates in the matched sample to those with adolescent depression.

**Figures S4.** Balance plots displaying the association between the confounders and the instrument use for the one- and two-year treatment window in those with adolescent depression.

Fluoxetine

Citalopram

Sertraline

Escitalopram


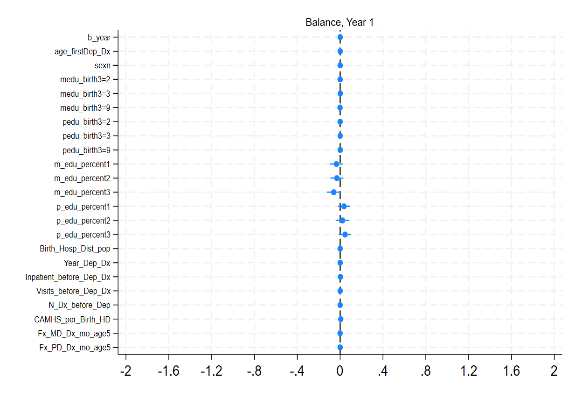

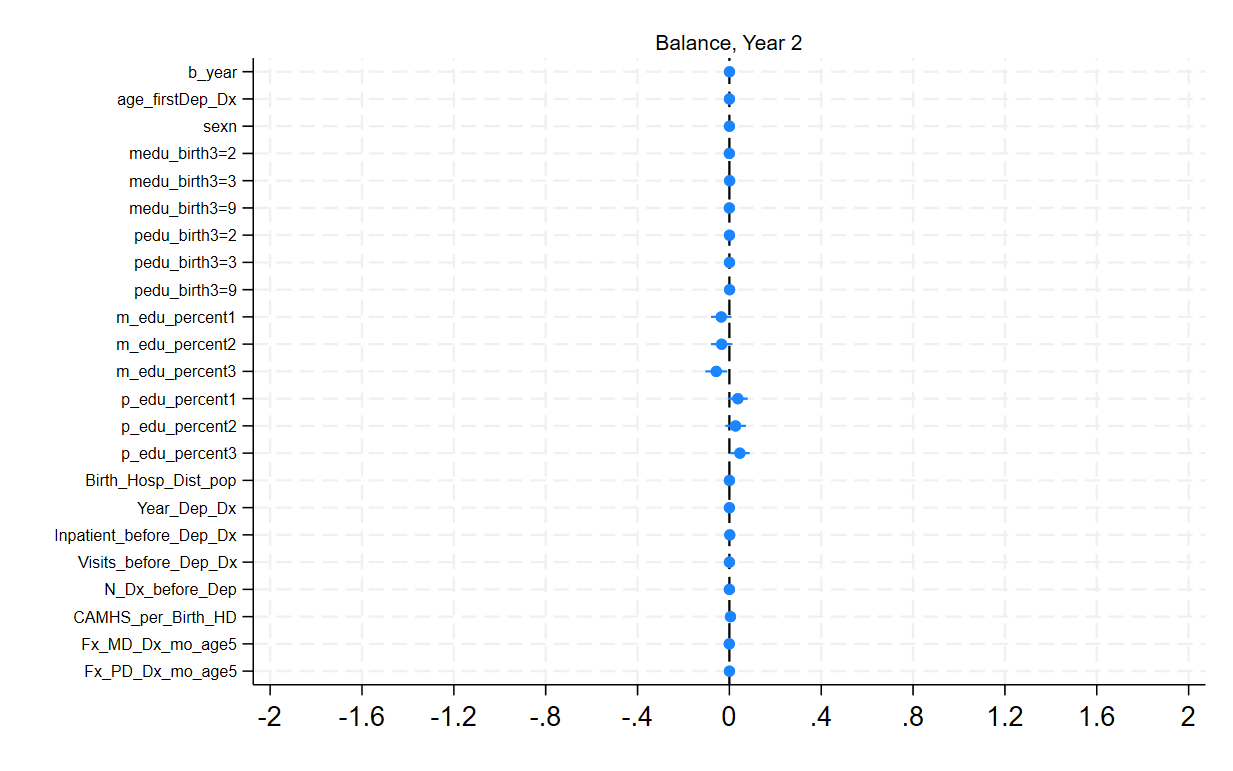

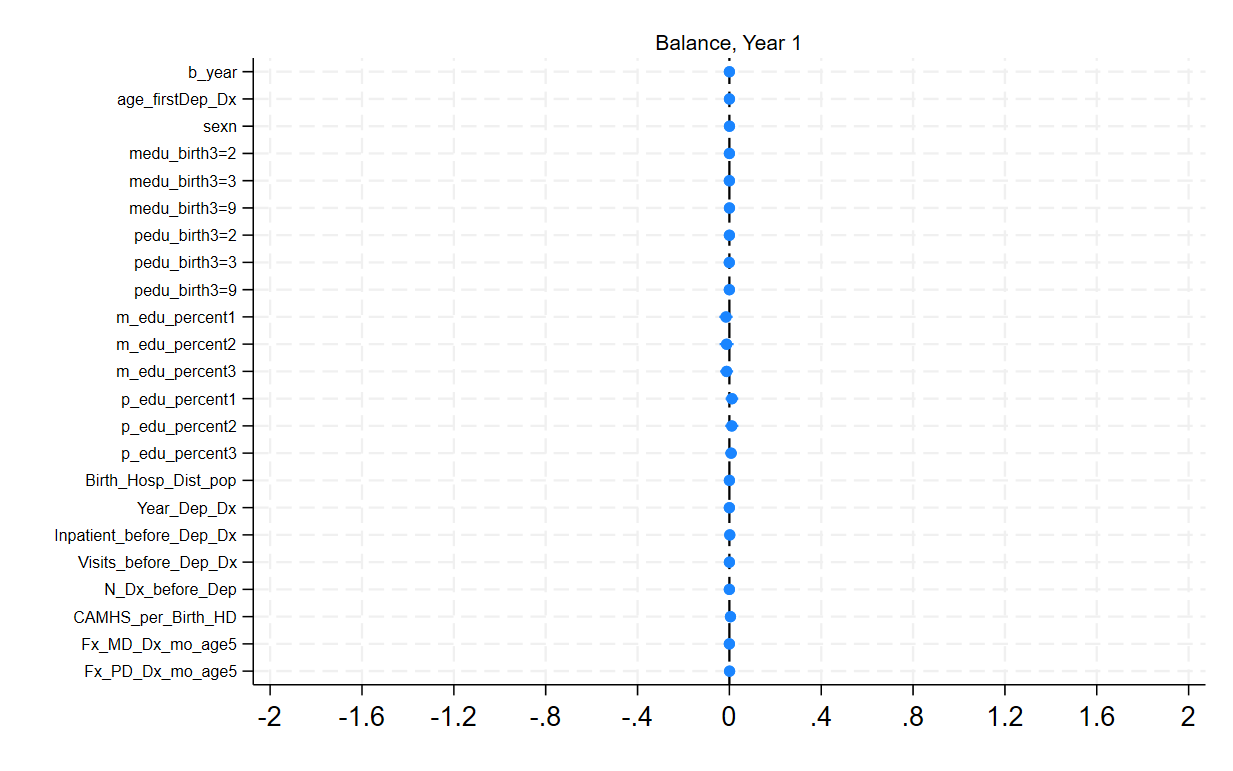

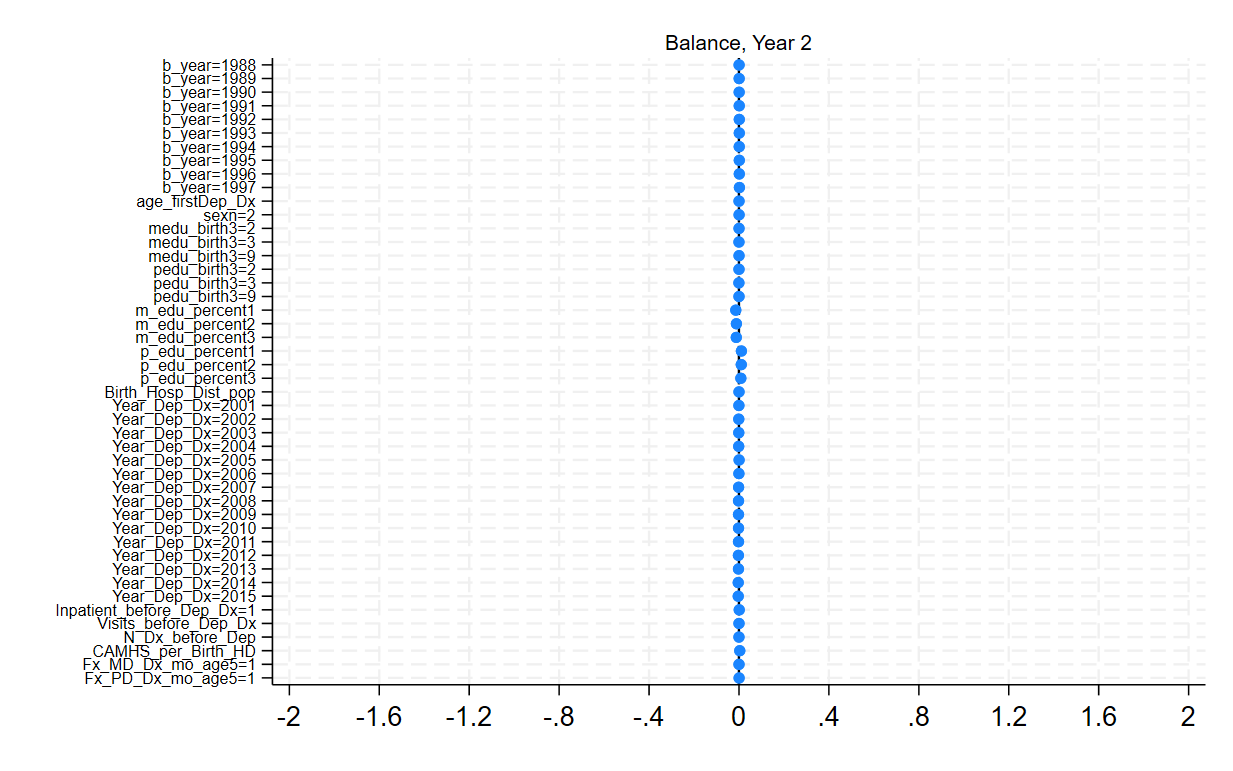

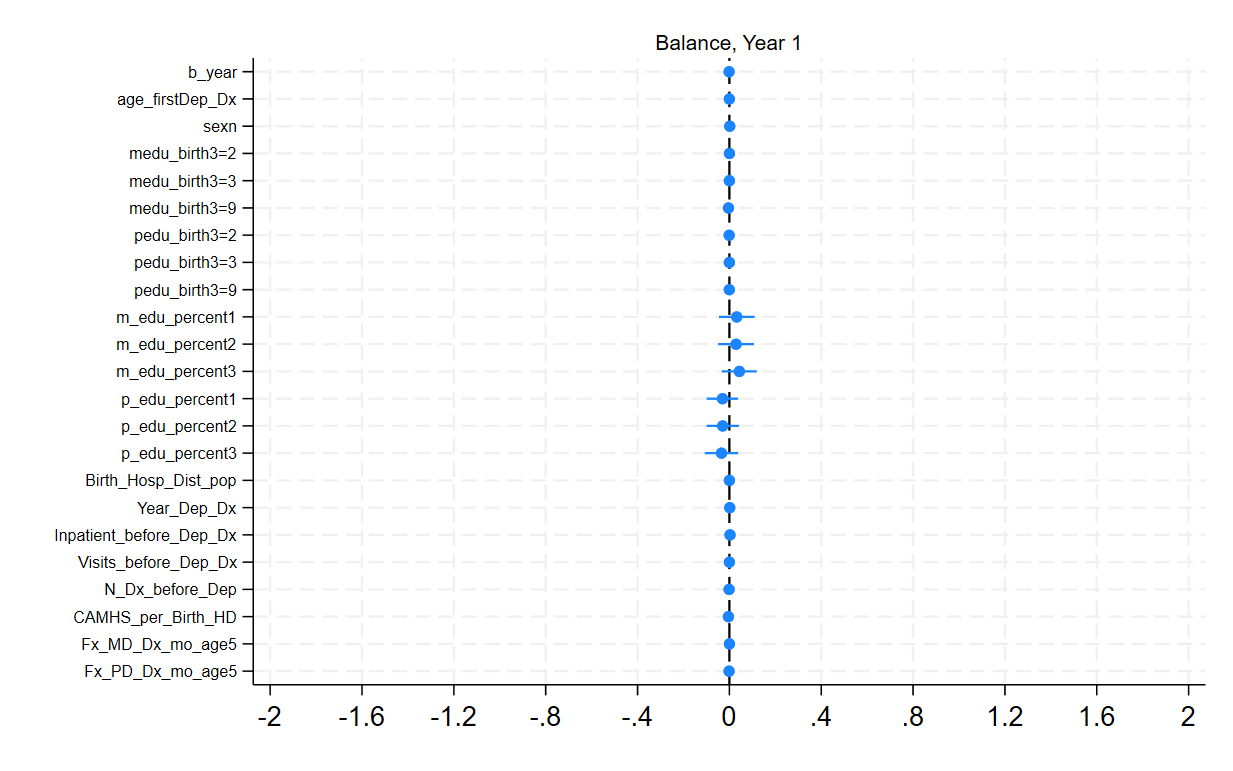

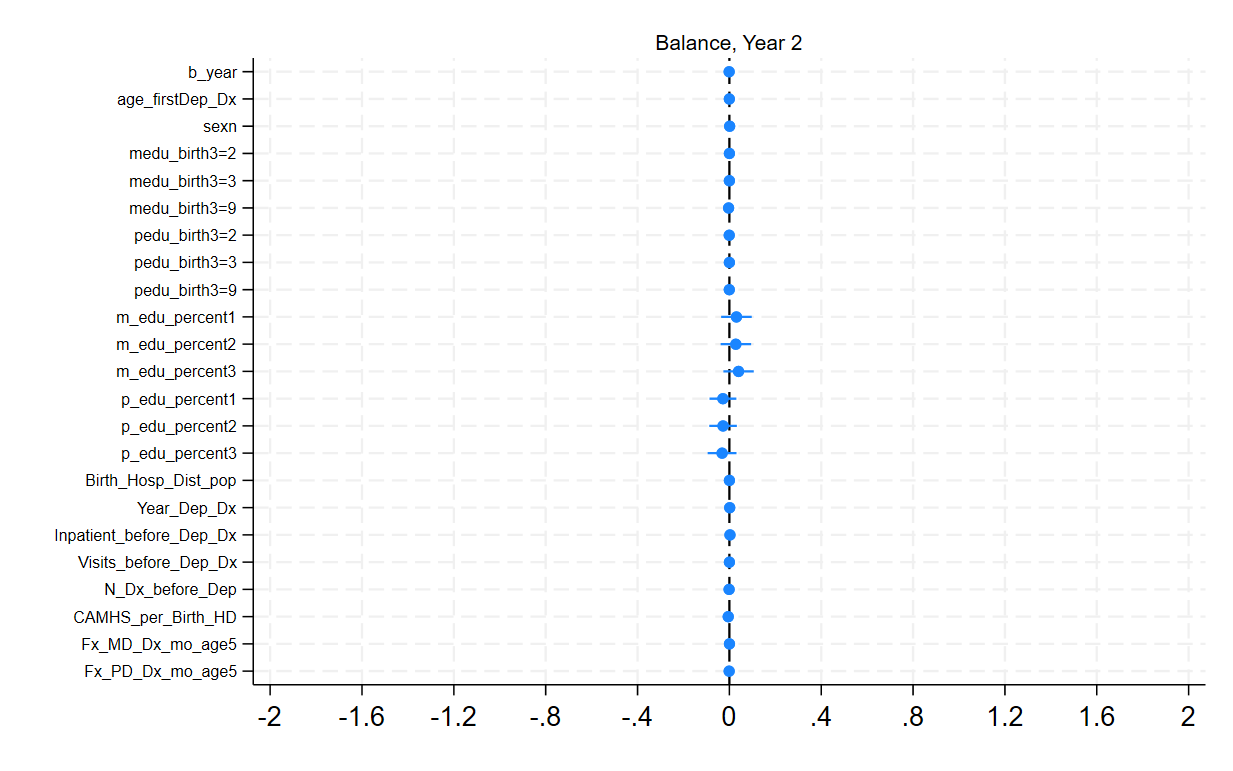

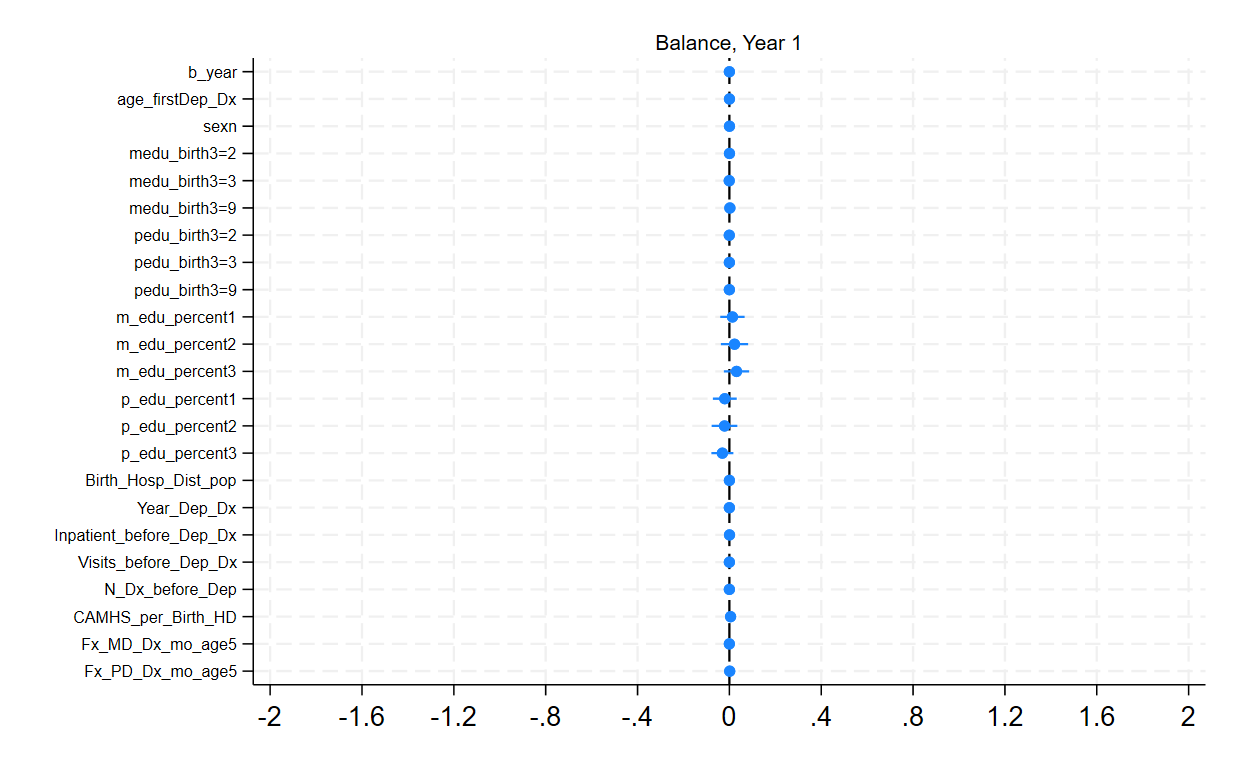

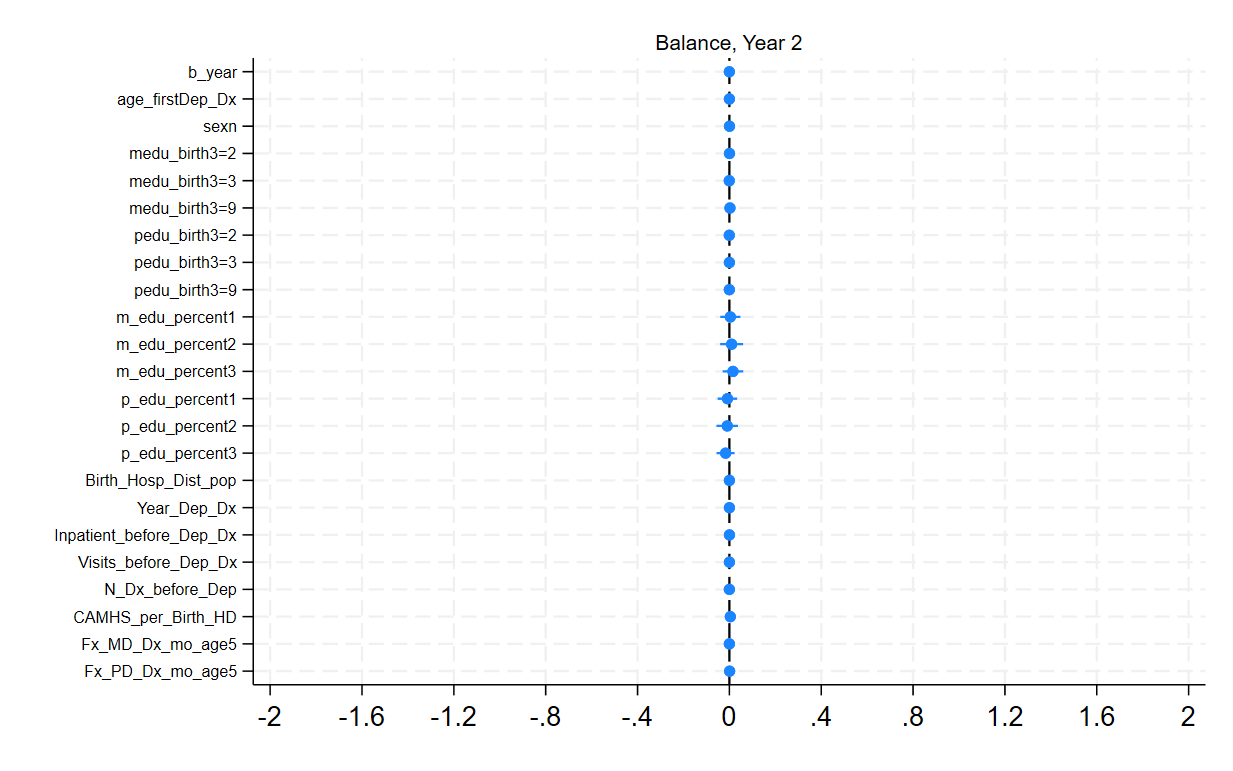


**Figure S5.** The hospital district variability in prescribing for those with depression across each of the years as well as medication trajectories stratified into tertiles.


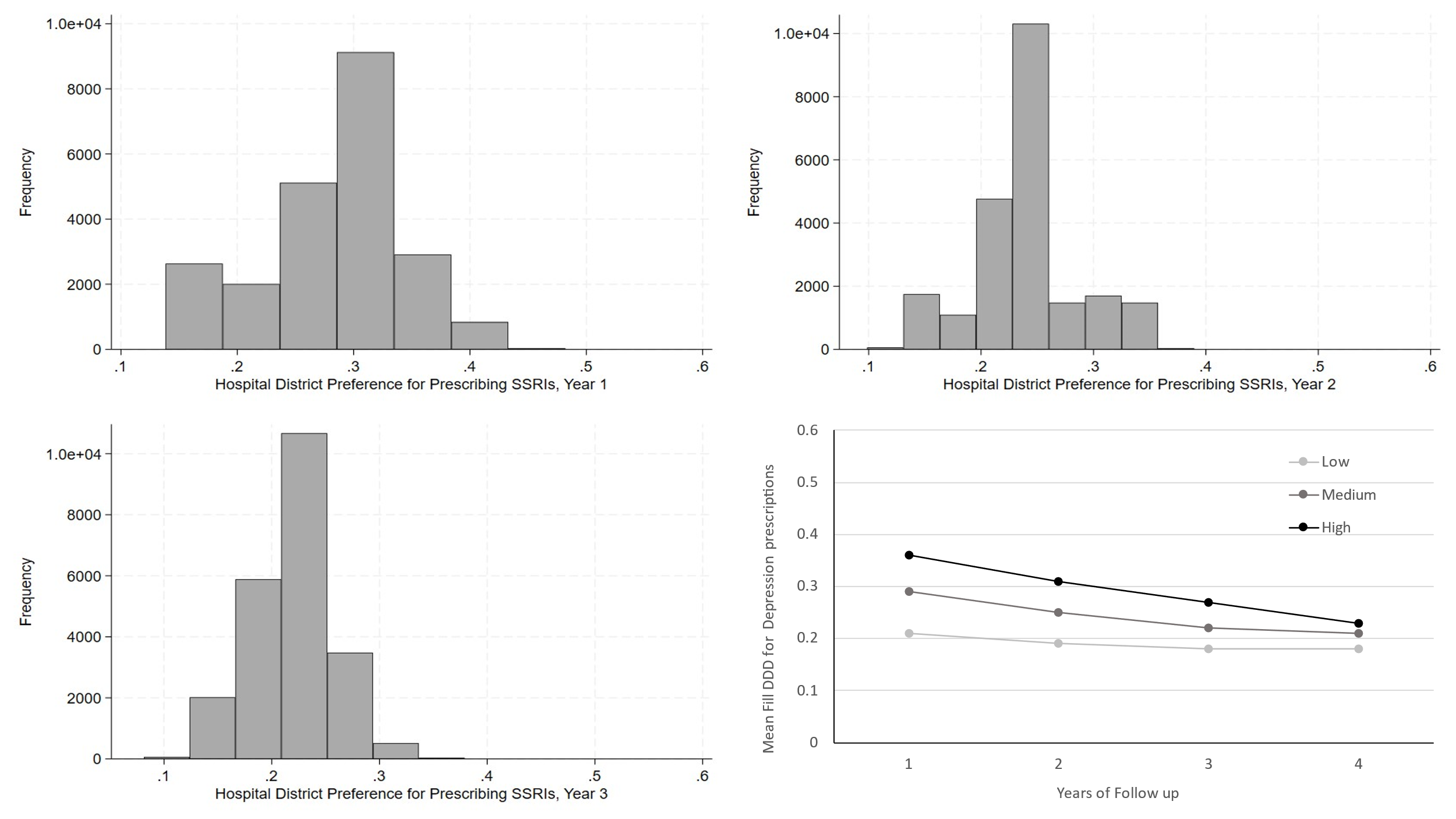

Supplement: Healy et al. supplementary material [file S0924933825100503sup001.docx]
